# Differential immune landscapes in appendicular versus axial skeleton

**DOI:** 10.1371/journal.pone.0267642

**Published:** 2022-04-27

**Authors:** Aqila A. Ahmed, Michael J. Strong, Xiaofeng Zhou, Tyler Robinson, Sabrina Rocco, Geoffrey W. Siegel, Gregory A. Clines, Bethany B. Moore, Evan T. Keller, Nicholas J. Szerlip

**Affiliations:** 1 Department of Internal Medicine, University of Michigan, Ann Arbor, Michigan, United States of America; 2 Biointerfaces Institute, University of Michigan, Ann Arbor, Michigan, United States of America; 3 Department of Neurosurgery, University of Michigan, Ann Arbor, Michigan, United States of America; 4 Department of Urology, University of Michigan, Ann Arbor, Michigan, United States of America; 5 Department of Orthopaedic Surgery, University of Michigan, Ann Arbor, Michigan, United States of America; 6 Veterans Affairs Medical Center, Ann Arbor, Michigan, United States of America; 7 Department of Microbiology and Immunology, University of Michigan, Ann Arbor, Michigan, United States of America; Universita degli Studi di Parma, ITALY

## Abstract

Roughly 400,000 people in the U.S. are living with bone metastases, the vast majority occurring in the spine. Metastases to the spine result in fractures, pain, paralysis, and significant health care costs. This predilection for cancer to metastasize to the bone is seen across most cancer histologies, with the greatest incidence seen in prostate, breast, and lung cancer. The molecular process involved in this predilection for axial versus appendicular skeleton is not fully understood, although it is likely that a combination of tumor and local micro-environmental factors plays a role. Immune cells are an important constituent of the bone marrow microenvironment and many of these cells have been shown to play a significant role in tumor growth and progression in soft tissue and bone disease. With this in mind, we sought to examine the differences in immune landscape between axial and appendicular bones in the normal noncancerous setting in order to obtain an understanding of these landscapes. To accomplish this, we utilized mass cytometry by time-of-flight (CyTOF) to examine differences in the immune cell landscapes between the long bone and vertebral body bone marrow from patient clinical samples and C57BL/6J mice. We demonstrate significant differences between immune populations in both murine and human marrow with a predominance of myeloid progenitor cells in the spine. Additionally, cytokine analysis revealed differences in concentrations favoring a more myeloid enriched population of cells in the vertebral body bone marrow. These differences could have clinical implications with respect to the distribution and permissive growth of bone metastases.

## Introduction

The spine represents one of the most common and debilitating sites of metastatic spread of malignant disease [1–5]. In the U.S., roughly 300,000–400,000 people live with bone metastases [6, 7]. About two-thirds of all symptomatic bone metastases are located in the spine, with up to 74% of patients having spine metastases at autopsy regardless of tumor histology [4]. The median survival for cancer patients after the diagnosis of bone metastases is only 2–3 years, even with aggressive treatment [8, 9]. Unfortunately, as a result of tumor growth, spinal cord and nerve compression ensues, and spinal metastases are large contributors to cancer pain and disability for these patients [10]. Molecular mechanisms guiding preferential metastatic growth in individual vertebral bodies are still unknown. Current theories postulate that the presence of red marrow in adult vertebrae, and the existence of vertebral venous plexuses, devoid of valves, may explain the high incidence of spinal metastases [11]. Another leading theory is Paget’s “Seed and Soil” hypothesis, which proposes that the colonization and growth of malignant cells (i.e., “seed”) are determined by their ability to migrate to and proliferate in the new distant environment (“soil”) where they now reside [12]. This phenomenon may be due to the presence or lack of local factors that either stimulate or inhibit growth, as seen analogously in ecological models of invasive species.

Despite being highly heterogeneous diseases at the cellular, genetic, transcriptomic, and micro-environmental levels, many different primary cancers have a tendency to metastasize to the spine with very few mutations that are similar across cancers [13]. Taking this into consideration, the argument can be made that either multiple mutations are serving the same purpose and causing the cancer to spread to and grow preferentially in the spine or this predilection is related to something else not entirely inherent to the tumor, but to the location itself. Theoretically, differences in the local bone microenvironments of the appendicular skeleton (long bones) and the axial skeleton (spine) may account for this predilection. This notion favors the “soil” argument.

Furthermore, the bone houses the bone marrow, which is a dynamic organ composed of protein, water, fat, and heterogeneous cell populations of hematopoietic precursors that mature to replenish the immune system [14, 15]. As such, there are continuous changes in the makeup of the cellular landscape within bone marrow with increasing age and responses to different environmental influences and health states [15, 16]. For example, at birth, there is tremendous metabolic activity within the spinal bone marrow, which throughout life gradually transitions to a less metabolically active marrow through a process known as marrow conversion [15, 17, 18]. Alternatively, various physiologic and pathologic conditions (e.g., obesity, cigarette smoking, athletic activity, and anemia) can influence the bone marrow to revert back to the more metabolically active marrow termed marrow reconversion, which is a patchy and asymmetrical process compared to normal conversion [19, 20].

Not surprisingly, the spine is the largest source of bone marrow in the body [14, 15, 21]. Additionally, alterations in the bone may have significant consequences for the immune system, thereby promoting tumor growth [22–25]. Studies show that immunodeficient patients have an increased incidence of various cancers, whereas a healthy immune system can prevent the development of cancer [24]. It has been shown that the bone is a somewhat immune-privileged site, where cells are better able to avoid immune surveillance [26, 27]. This exists because the bone has a dampened immune response to protect the critical hematopoietic stem cell compartment. The resulting microenvironment theoretically provides a niche for tumor cells to thrive and evade the host immune defense mechanisms as well as affects tumor response to immune therapies. Jiao et al. [28] reported that differences in the tumor microenvironment in bone lead to suboptimal response to immune check point therapy in certain cancer types.

Limited studies have examined this unique immune microenvironment. Most of these studies were performed predominantly on soft tissue and in the instance of bony examinations have been mostly localized to long bones [29, 30]. To the best of our knowledge, no study to date has extensively examined the bone marrow of different types of bone.

Mass cytometry by time-of-flight (CyTOF) is a relatively novel technology for single-cell proteomic analysis of the immune system. It enables almost limitless numbers of markers on individual cells by the use of monoclonal antibodies conjugated to rare earth metals (as opposed to fluorescent markers used with flow cytometry). CyTOF is highly specific with limited signal overlap, which allows for the detection of more markers per cell [31, 32]. This along with computational statistics allows for the distinction of cell types that were once impossible to distinguish. Here we utilized CyTOF to examine differences between the immune cell landscapes of the long bone and vertebral body bone marrow in both murine and human samples. Our analysis showed differential composition of immune cells residing within the various bone marrow niches. Further, this analysis revealed significant differences in myeloid subpopulations within the vertebral body compared to the long bone.

## Materials and methods

### Sample preparation

#### Human bone marrow cell preparation

Two of the four human bone marrow samples were collected at the University of Michigan under Institutional Review Board (IRB) protocol HUM00113360 and 2 samples were from the Ann Arbor Veteran’s Affairs Hospital under protocol IRB-2017-1029. Patients were matched (age within a decade, ethnicity, sex, medications in similar drug classes), and the specimens were stored in tubes containing 50 mM EDTA. The human vertebral body samples were obtained as bone marrow aspirates taken from the lumbar spine after drilling tracks for placement of pedicle screws. The human long bone samples were obtained as bone marrow aspirates taken from hip replacement patients. Upon receiving specimens, red blood cells were lysed with 1X ACK lysis buffer, and bone marrow was strained though a 70-μm mesh filter. After centrifugation (300 g for 5 min) to pellet cells, cells were washed with DMEM+GlutaMAX/10% FBS media (Invitrogen, Carlsbad, CA, USA) and resuspended in ice-cold FBS/10% DMSO freezing media (DMSO: Sigma, St. Louis, MO, USA). Cells were counted using a hemocytometer (Thermo Fisher, Waltham, MA, USA) and frozen down 5–20 million cells/mL in freezing media and stored in a liquid nitrogen tank. When cells were needed for experiments, they were thawed in a 37°C water bath in DMEM+GlutaMAX/10% FBS media plus 5U Benzonase (Sigma) to prevent cell clumping. Cells were pelleted via centrifugation at 300 g for 5 min and supernatant was removed to minimized exposure to DMSO before proceeding with downstream experiments.

### Mouse spine and long bone dissociation to single cells

All animal procedures were conducted in accordance with the Guide for the Care and Use of Laboratory Animals at the University of Michigan and were approved by the Institutional Animal Care and Use Committee. Male C57BL/6J mice 10–14 wk old, strain #000664 (Jackson Laboratory, Bar Harbor, ME, USA), were housed in accordance with institutional guidelines, with no more than 5 mice per cage, 12-hour light and dark cycles, bedding changed weekly, and supplemented with enrichment materials. All mice were fed a standard diet.

Mice were euthanized via carbon dioxide overdose followed by cervical dislocation. The spine, tibias, and femurs of the mice were surgically removed and diced into small sections using a scalpel. Sections were added to 50-mL tubes containing bone marrow media (RPMI-1640 medium/10% FBS/1% L-Glutamine; Invitrogen). Samples were vortexed vigorously and strained through a 70-μm mesh filter. Bone marrow media was added on the filter to wash the remaining bone sections. Cells were pelleted via centrifugation at 350 g for 5 min and supernatant was removed. Red blood cells were lysed with 1x ACK lysis buffer and samples were centrifuged at 350 g for 5 min. Cells were resuspended in FBS/10% DMSO freezing media. Cells were counted using a hemocytometer (Thermo Fisher) and frozen down 5–10 million cells/mL in freezing media and stored in a liquid nitrogen tank. When cells were needed for experiments, they were thawed in a 37°C water bath and DMEM+GlutaMAX/10% FBS media was added. Cells were pelleted via centrifugation at 300 g for 5 min and supernatant was removed to minimize exposure to DMSO before proceeding with downstream experiments.

### Antibodies for mass cytometry

Pre-conjugated antibodies for mass cytometry were obtained (Fluidigm, South San Francisco, CA, USA, or Harvard Mass cytometry core). Antibodies used in mice were titrated and validated in house. Both mouse and human panels are shown in **S1 and S2 Tables**, respectively.

### Staining of cells with metal-tagged antibodies

Cell-ID Cisplatin-195Pt and Cell-ID Intercalator Iridium-191/193 (Fluidigm) were used to identify live cells. The cells were washed once with 1x Maxpar® PBS (Fluidigm) by pelleting at 300 g for 5 min at room temperature and stained with 1.25 μM live/dead stain (Cell-ID Cisplatin-195Pt diluted Maxpar PBS from 500-mM stock) at room temperature for 5 min. Free cisplatin was quenched by washing the cells with Maxpar staining buffer (Fluidigm). The cells were then incubated with TruStain FcX (anti-mouse CD16/32, BioLegend^®^, San Diego, CA, USA) for 10 min at room temperature to block the Fc receptors. For cell surface marker staining the metal-tagged antibody cocktail was made in Maxpar staining buffer and added to the cells in the presence of TruStain FcX (BioLegend^®^) and incubated on ice for 40 min. Following cell surface marker staining, the cells were washed twice with Maxpar staining buffer and fixed with fresh 1.6% paraformaldehyde in Maxpar PBS for 20 min at room temperature. Finally, stained cells were incubated with Iridium DNA intercalator (Fluidigm) in Maxpar fix and perm buffer (Fluidigm) for up to 48 h at 4°C. The samples were acquired using a CyTOF Helios system (Fluidigm), maintained and tuned according to the manufacturer’s instructions. In addition, internal vendor-set calibration was performed before acquiring samples. The fixed cells were washed twice with PBS, resuspended in Maxpar cell acquisition solution (Fluidigm), and filtered through 40-μM cell strainer. Recommended concentrations of EQ four element calibration beads (Fluidigm) were added to the samples before acquiring them on CyTOF. The samples were acquired on CyTOF at approximately 100–400 events/sec using WB injector (Fluidigm). The EQ four element calibration beads were used to normalize data using a bead-based passport specific to the manufactured bead lot.

### CyTOF data analysis

Manual gating for live CD45+ singlets in each sample was performed using Cytobank [33]. Flow cytometry standard files were than exported from Cytobank gates and later analyzed using the cytofkit R package [34] (https://bioconductor.org) run in RStudio v.1.0.44 (2016-11-01) (RStudio, Boston, MA, USA). PhenoGraph clustering [35] was performed using all markers on a fixed number of 5,000 cells without replacement from each file and combined for analysis. Resulting *t*-SNE plots were subsequently filtered by marker expression and bone marrow group to visualize differences between spine and long bone. Major cell subpopulations were annotated based on prior knowledge of expected marker expression in various cell types and are illustrated in **Table 1** for mouse and **Table 2** for human.

**Table 1:** Mouse CyTOF immunophenotyping.

Cell types	Markers
Monocytes	CD11b+, LY-6C+,
Classical monocytes	LY-6C^hi^, CD11b+, CD43+
Monocytes/macrophages	Ly6C^lo^, Cd11b+, CD43^hi^
Cd8a+ DC	CD11c+, CD11b^lo^, MHCII+, CD8+
Memory T helper cells	CD3+, CD4+, CD26L+
T helper cells	CD3+, CD4^hi^, FR4^hi^
TCRgd+ T cells	CD3+, TCRgd+
Naïve T cells	CD3+, CD4+, CCR7+, CD62L^lo^
NK T cells	CD11b+, NK1.1+, CD335+, CD3-
Activated B cells	B220+, MHCII+
B cells	CD45R, B220+, CD19+
Eosinophils	CD11b+, Ly6G+, Siglec F+, CD43+
Granulocytes	CD11c-, Cd11b+, Ly6G+, Ly6C^lo^
pDC	CD11c+, CD11b^lo/-^, MHCII^lo^, B220
cDC	CD11c^hi^, CD11b+, MHC II^hi^, CD8^lo/-^
Mast cells	CD117+, FcεRIα+
MDSCs	Ly6G+, Ly6C^lo^, Cd11b+
Granulocytic-MDSCs	CD11b^hi^, Ly6G^hi^, Ly6C^lo^
Myeloid progenitors	Sca-1+, CD117^hi^, CD150-, CD3-, B220-, Cd19-, Cd11b-,

**Table 2:** Human CyTOF immunophenotyping.

Cell types	Markers
Classical monocytes	CD45+, CD19-, CD3-, CD14+, CD16-
Non-classical monocytes	CD45+, CD19-, CD3-, CD14+, CD16+
Naive B cells	CD45+, CD14-, CD16-, CD161-, CD19+, CD3-, CD20+, CD27-, IgD+
Transitional B cells	CD45+, CD14-, CD16-, CD161-, CD19+, CD3-, CD20+, CD24+, CD38+
pDC cells	CD45+, CD14-, CD20-, CD19-, CD3-, HLADR+, CD56-, CD16-, CD123+, CD11C-
DC cells	CD45+, CD14-, CD20-, CD19-, CD3-, HLADR+, CD56-, CD16-, CD123-, CD11C+
NK cells	CD45+, CD14-, CD20-, CD19-, CD3-, CD56+, CD161+, CD123+, CD16+/-
NK T cells	CD3+, CD28+, CD161+
Effector memory CD8 T cells	CD45+, CD14-, CD20-, CD3+, TcRgd-, CD4-, CD8+, CCR7-, CD45RO+, CD45RA-
Terminal effector CD8 T cells	CD45+, CD14-, CD20-, CD3+, TcRgd-, CD4-, CD8+, CCR7-, CD45RO-, CD45RA+
Naive CD8 T cells	CD45+, CD14-, CD20-, CD3+, TcRgd-, CD4-, CD8+, CCR7+, CD45RO-, CD45RA+
Activated CD4 cells	CD4+, HLA-DR+, CD38+
Central memory CD4 T cells	CD45+, CD14-, CD20-, CD3+, TcRgd-, CD4+, CD8-, CCR7+, CD45RO+, CD45RA-
Effector memory CD4 T cells	CD45+, CD14-, CD20-, CD3+, TcRgd-, CD4+, CD8-, CCR7-, CD45RO+, CD45RA-
Naive CD4 T cells	CD45+, CD14-, CD20-, CD3+, TcRgd-, CD4+, CD8-, CCR7+, CD45RO-, CD45RA+
Treg	CD45+, CD14-, CD20-, CD3+, TcRgd-, CD4+, CD8-, CD25+, CD127-, CCR4+, HLA-DR+
Polymorphonuclear leukocytes	CD66b+, CD16+
Myeloid progenitor cells	CD45RA+, CD38+, CD14^lo^

### Cytokine analysis

Murine bone marrow cells from the vertebral body and long bone were sent to Eve Technologies (Calgary, AB, Canada), where a commercial murine cytokine assay (Mouse Cytokine Array / Chemokine Array 44-Plex (MD44)) was utilized for data analysis. Data were populated in an Excel (Microsoft; Redmond, WA, USA) spreadsheet and displayed as concentrations (pg/mL). Cytokine concentrations were normalized to the number of cells per sample. Samples were sent in triplicate.

### Statistical analysis

Frequencies for each population were exported to Excel and GraphPad Prism 6 (GraphPad Software Inc., La Jolla, CA, USA) for subsequent analysis and data presentation. Statistical analysis between groups was performed using a paired Student’s t test. Statistical significance was set at *p* < 0.05.

## Results

### The immune cell landscapes of murine long bone and vertebral body bone marrow differ

**Fig 1:**
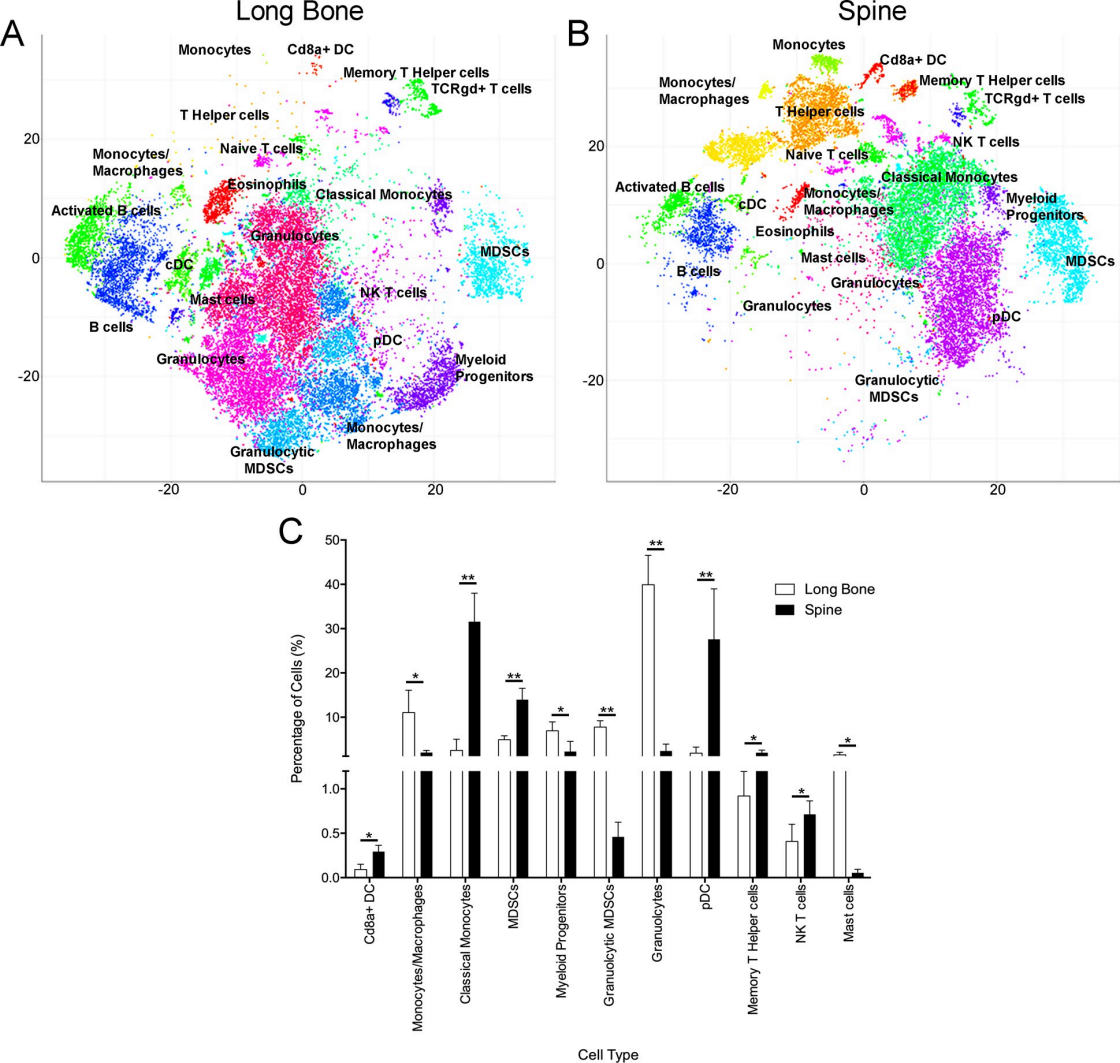
Characterization of the immune microenvironments of mouse vertebra and femur bone marrow using CyTOF. Representative t-SNE plots show PhenoGraph clusters for (**A**) long bone and (**B**) spinal bone marrow. (**C**) Frequencies of statistically different immune cell populations between the two bone marrow samples. All cell types listed were statistically significant; *p* < 0.05. n = 5, percent of cells (SD, * denotes *p* < 0.05, ** denotes *p* < 0.001).

To examine the native immune cell populations within the bone marrow microenvironment of long bones and vertebral bodies, we employed CyTOF immunophenotyping, which is a relatively novel technology for single-cell proteomic analysis of the immune system. As previously stated, the advantage of CyTOF is the ability to utilize many surface markers at once without the overlap that results with fluorescence. Combining the ability to probe over 100 metal-conjugated antibodies and high-dimensional analysis allows one to better distinguish the immune composition of bone marrow. Analysis of the murine bone marrow cells harvested from murine femur (n = 5) and vertebral bodies (n = 5) using CyTOF demonstrated a statistically significant increase in MDSCs, classical monocytes, CD8a+ dendritic cells, plasmacytoid dendritic cells, memory T helper cells, and NK T cells in the vertebral body compared to the long bone. In contrast, we observed a statistically significant increase in granulocytes, granulocytic MDSCs, monocytes/macrophages, myeloid progenitor cells, and mast cells in the long bone marrow compared to the vertebral body bone marrow (**Fig 1**).

### The immune cell landscapes of human long bone and vertebral body bone marrow differ

**Fig 2:**
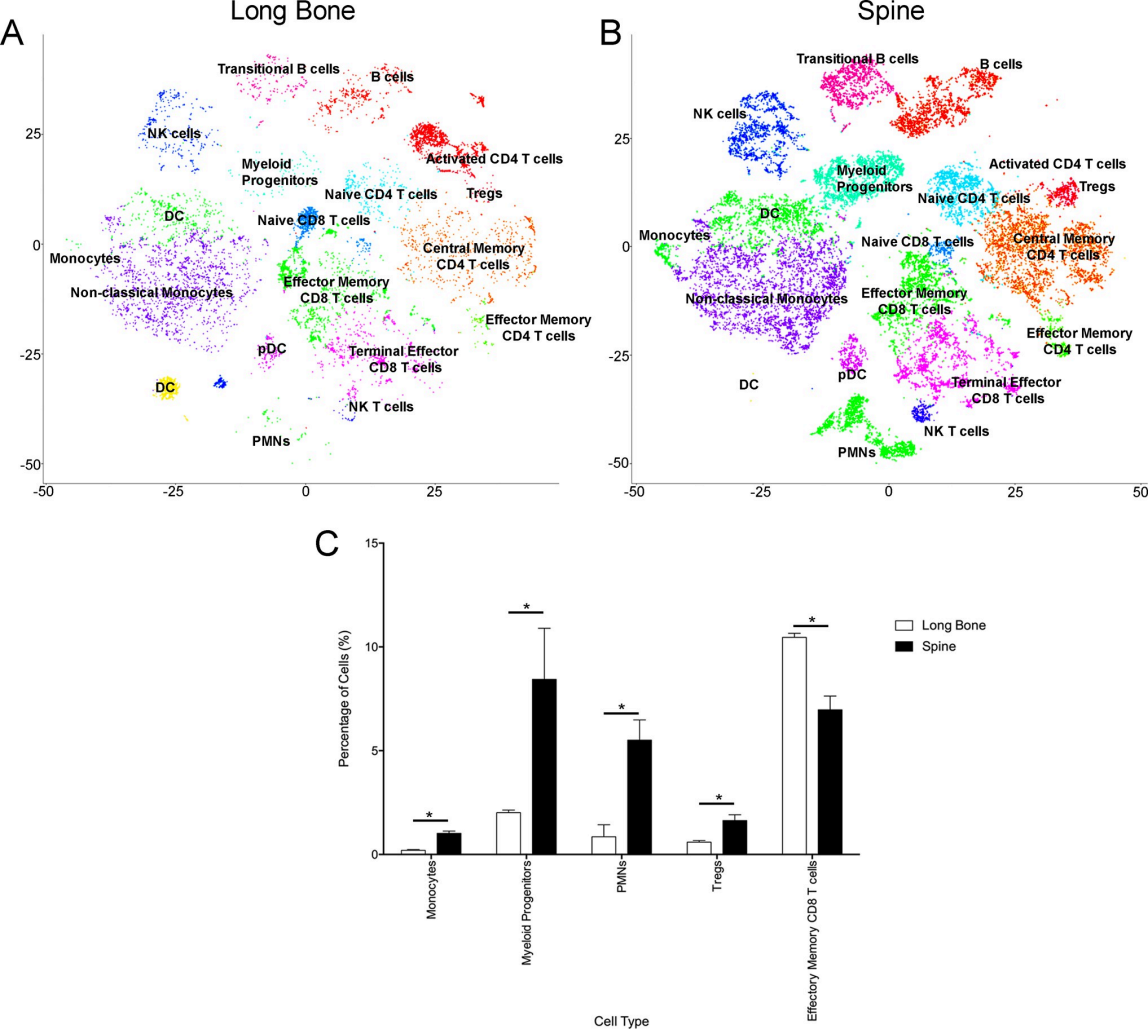
Characterization of the immune cell landscapes of human long bone and vertebral body bone marrow using CyTOF. Representative t-SNE plots show PhenoGraph clusters for (**A**) long bone and (**B**) spinal bone marrow. (**C**) Frequencies of statistically different immune cell populations between the two bone marrow samples. All cell types listed were statistically significant; *p* < 0.05. n = 4, percent of cells (SD, * denotes *p* < 0.05, ** denotes *p* < 0.001).

To determine if these differences between murine samples held true in human bone marrow, we employed our CyTOF pipeline for the analysis of human femurs and lumbar vertebral bodies. Each sample came from a different patient, and patients were all white males aged 57–61 years old. Additionally, medications were matched controlled as much as possible with overall similar drug classes, paying particular attention to medications known to affect bone (**S3 Table**). After controlling for these variables, four human bone marrow samples, obtained from two femurs and two lumbar vertebral bodies (patients without cancer undergoing surgeries for degenerative disease of either the hip or spine, respectively), were selected for analysis using a commercially available CyTOF immunophenotyping panel. Based on surface marker expression, we identified at least 10 CD45+ populations, including major lymphocyte and myeloid cell subsets that were statistically significantly different between spine and long bone marrow. Certain other populations showed dramatic differences; however, the variance between the individual samples was too high to reveal any significance. On the other hand, monocytes, myeloid progenitor cells, regulatory T cells (Tregs), and polymorphonuclear leukocytes were significantly increased in the human vertebral body compared to the long bone (**Fig 2**). In addition, there was a statistically significant increase in effector memory CD8 cells in the human long bone compared to the vertebral body (**Fig 2**). Overall, our data suggest that the global shifts in monocytes and myeloid-derived cells are conserved across species, resulting in what we believe may be a more immunosuppressive environment within the vertebral body bone marrow compared to the long bones.

**Fig 3:**
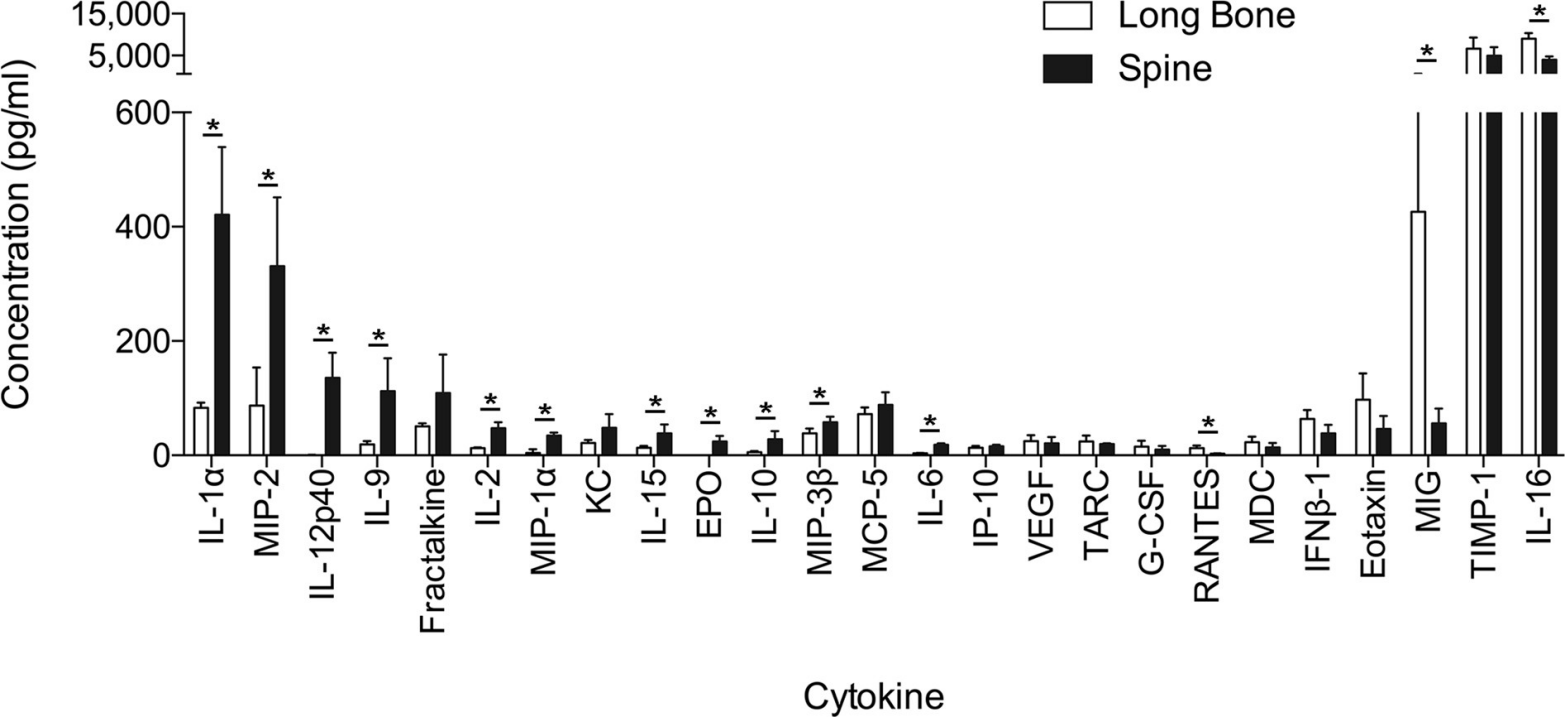
Differential expression of cytokines is based on bone marrow location. Forty-four mouse cytokines were analyzed between murine vertebral body and long bone. Values with concentrations >10 pg/mL are displayed as mean ± SD. Statistical significance equals *p* ≤ 0.05.

To further characterize the differences in the bone marrow microenvironments, we examined the cytokines derived from bone marrow cells from murine spine and long bone. Based on this analysis, we identified several cytokines that are heavily involved in myeloid cell chemotaxis (IL-10, IL-6, MIP-2/CXCL2) and were statistically significantly increased in the spine compared to long bone (**Fig 3**). There was a trend toward increased IL-13 in the spine, but this did not reach statistical significance (**S1 File**). In addition, while IL-17 was statistically significantly increased in the spine compared to the long bone, the concentrations (3.5 pg/mL and 0.39 pg/mL, respectively) failed to reach our threshold of 10 pg/mL (**S1 File**). Other statistically significant cytokines that were increased in the spine include IL-1α, IL-12p40, IL-9, IL-2, MIP-1α/CCL3, IL-15, and MIP-3β/CCL19 (**Fig 3**). In the long bone, there was a statistically significant increase in RANTES, MIG/CXCL9, and IL-16 compared to the vertebral body (**Fig 3**).

## Discussion

The spine represents one of the most common and debilitating sites of metastatic spread of primary cancer [1–5]. However, the increased propensity for spinal metastases as opposed to long bone metastases is still unclear. Furthermore, in-depth genomic analysis of several human malignancies has revealed very few common mutations among bone metastatic cancer histologies, suggesting that not only are there multiple and specific oncogenic pathways that are employed by primary cancers to achieve metastatic spread [13] but that there are likely other inherent factors outside the primary tumor’s microenvironment that permit such metastatic growth. As such, differences in the local bone microenvironments of the appendicular skeleton (long bones) and the axial skeleton (spine) may account for cancer preference to the spine. In this study we aimed to obtain an understanding of any inherent differences in the native bone marrow of vertebral bodies and long bones by examining bone marrow in mice/humans without cancer. We demonstrate that there are baseline differences in the immune landscape between long bone and spine bone marrow, with a significant increase in the MDSC type in the murine spine bone marrow compared to long bone. Furthermore, differences in the cytokine profiles suggest differences in native milieu between the spine and long bone marrow.

The localization of a heterogeneous population of immune cells at various levels of maturation with minimal differences in cell surface markers can mask the identification of unique cell populations. A specific example of this are the similarities between many mature neutrophils and MDSC subtypes [36]. The spectral overlap of traditional flow cytometry methods limits the number of cell surface markers that can be analyzed simultaneously. Therefore, we employed CyTOF immunophenotyping as an alternative approach to help delineate this uniquely complex and heterogenous bone marrow microenvironment through the use of rare earth metals and mass cytometry, which all but eliminate spectral overlap issues [37, 38].

Interestingly, in the murine long bone, we observed an increase in granulocytes, granulocytic MDSCs, and monocyte-macrophages compared to the murine spine, whereas in the murine spine bone marrow, we observed an increase in MDSCs and classical monocytes. It is possible that some of the cells classified as granulocytic MDSCs were misclassified and may represent granulocytes. Despite using CyTOF immunophenotyping with the vast array of surface marker antibodies, there still may be overlap between these various cell types and misclassification would not be unreasonable. Further, the myeloid progenitor cell type, which includes precursors to red blood cells, platelets, granulocytes, monocyte-macrophages, dendritic cells, and mast cells, was also increased in the murine long bone. To further characterize cell populations functional experimentation would be important.

However, when compared to the human samples, myeloid progenitor cells were elevated in the human vertebral body bone marrow compared to the human long bone. This discrepancy in observations can be partly accounted for by differences in species and characterization. Specifically, MDSC surface markers are better defined in the murine population compared to human. Another factor that may account for this discrepancy that is gaining more attention is the effects of sex and age on the immune system [39]. While sex was controlled for in our study by analyzing only male mice and human subjects, the impact of age may have influenced the differences in immune cell populations between our mouse and human CyTOF profiles. In our analysis, age would not completely factor into the intraspecies comparisons between long bone and vertebral body bone marrow since age-specific changes in immune profiles would be consistent throughout the mouse. Lastly, the human bone marrow samples were obtained from patients with degenerative osteoarthritis, which may have also contributed to the differences in immune cell populations between the human and murine samples. Specifically, there is mounting evidence supporting the role of low-grade inflammation in the pathophysiology of osteoarthritis [40, 41].

Numerous studies have reported the importance of the tumor microenvironment in facilitating metastatic dissemination and proliferation of cancer cells [24, 42–44] and have highlighted the role of the immune system in promoting or preventing tumor growth [22–24, 45–47]. Studies show that immunocompromised patients have an increased incidence of a variety of cancers, whereas a healthy immune system can prevent the development of cancer [24]. It is known that the local immune system plays an important part in the formation and propagation of bone metastases, but the specific cell types and mechanisms involved have not been investigated in different bone types.

Tumor cells have been shown to modulate the immune response due in large part to MDSCs. MDSCs are immature myeloid cells that represent a heterogeneous population consisting of precursors of granulocytes, macrophages, and dendritic cells that have potent immune suppressive activity [48–53]. These cells have been extensively studied in both murine and human and can be grouped into two subtypes, although other subtypes have been proposed, including early-stage MDSCs that lack both macrophage and granulocyte markers [48, 51, 54–56]. The MDSC population in humans has not been as well defined as the murine, with no standardized markers, although Bronte et al. [48] proposed a set of surface markers that could be used to help define the various subtypes of MDSCs but stated that functional assays to measure suppressive activity would have to be performed as well to fully define these cells.

Nevertheless, MDSCs are key regulators in the development of the premetastatic niche [57]. There is also growing evidence to suggest that MDSCs isolated in tumors have a different functional phenotype and that the ratios of different types of MDSCs influence the level of immune suppression [51]. These different cell types are very hard to distinguish by markers, no matter how many markers are utilized, and new studies rely on a combination of multiple surface markers and transcriptional analysis to fully delineate cell types [58, 59]. Further, there appear to be differences in the mechanisms regulating MDSC function in tumors and peripheral lymphoid organs, such that inhibition of STAT3 in tumor-bearing mice resulted in depletion of MDSCs in spleens but not in tumors [60]. Most of these studies have been conducted in soft tissue and peripheral lymphoid organs. Additionally, MDSCs have been shown to be upregulated in the bone marrow of the long bones of cancer patients, [61–63] and growing evidence suggests that these cells are important in driving the progression of cancer by suppressing both the innate and the adaptive immune responses [64].

Several cytokines have been implicated in the development and function of MDSCs, including MIP-2/CXCL2, IL-6, IL-10, IL-13, GM-CSF, and G-CSF [48, 65–68]. In our study, we demonstrate that MIP-2/CXCL2, IL-6, and IL-10 are significantly increased in the spine bone marrow compared to the long bone. Furthermore, there was a trend toward increased IL-13 in the spine. Although the increased concentration of these cytokines would suggest a more favorable microenvironment for MDSCs established in the spine compared to long bone, additional validation experiments are required. However, given these native differences in microenvironments, this fosters a potential preferential metastatic niche that should be further explored. Interestingly, in our previous work analyzing the effects of dura, which is in close proximity to the vertebral body, on tumor growth, we demonstrated that conditioned media from dural fibroblasts increased the growth, migration, and invasion of prostate cancer through the CXCR2 pathway [69]. While not significant, KC/CXCL1 was shown to be increased in the spine compared to the long bone (**S1 File**) in addition to an increase in CXCL2, both of which act on the CXCR2 pathway [70].

While not demonstrating statistical significance, there was a trend toward increased expression in the spine for Fractalkine/CX3CL1 compared to long bone. This cytokine has been demonstrated to be one of the most expressed chemokines in the central nervous system and is involved in inflammation and cancer [71, 72]. Additionally, Volin et al. [73] demonstrated an angiogenic effect of Fractalkine in rheumatoid arthritis. Fractalkine receptor has been demonstrated to be expressed in several cancer types (e.g., prostate, pancreas, breast carcinoma, glioma, and neuroblastoma), and this pathway has been demonstrated to play a role in tumorgenesis and metastasis [72]. These studies would suggest that although Fractalkine is involved with proinflammatory activation, its role is complex and governed by a multitude of factors.

In the long bone marrow, there appears to be the establishment of a less immunosuppressive environment compared to the spine. MIG/CXCL9, a known MDSC suppressive chemokine, was significantly increased in the long bone compared to spine [67]. Additionally, Thakur et al. [67] observed that increased levels of MIG/CXCL9, IP-10/CXCL10, and IFN-γ along with low levels of IL-6 and IL-1β, conditions we observed in the long bone marrow, implying a reduction of MDSCs. Finally, in a murine colorectal cancer model, Chen et al. [74] demonstrated that inhibition of IL-17 downregulated T cell infiltration through the modulation of CXCL9 and CXCL10. IL-17 has also been implicated in promoting the development of MDSCs [75, 76]. As mentioned previously, IL-17 was statistically significantly decreased in the long bone compared to the spine, which suggests decreased inhibition on CXCL9/10 and reduced development of MDSCs. Of note, although, IL-17 was statistically significant, the concentrations were low and additional validation experiments are needed to confirm this observation.

This study has several limitations. The human CyTOF panel was not set up to look at all the same cells as the murine panel, although there is ample overlap. The MDSC population could not be examined in human bone marrow specifically, although myeloid cells as a whole were examined. Future studies with additional human samples and panels designed to better examine differences in human MDSCs are planned. In addition, CyTOF may still miss differences. Ultimately, the question of cell population differences will have to be answered with a combination of surface markers, transcriptional analysis, and functional studies.

In summary, we demonstrate significant differences in not only the immune cell populations but also cytokines between the vertebral body and long bones both in murine and human bone marrow samples. These differences in immune cells in murine bone marrow were much more evident by using the CyTOF technique as compared to flow cytometry. To our knowledge, this is first study to compare the immune landscapes of the bone marrow of different bones. This study demonstrates a much more complicated and heterogeneous landscape than previously thought and challenges the scientific community to not group all bone marrow compartments together when conducting research. The pre-translational and clinical implications of the different immune landscapes in the initiation and growth of spinal metastases have yet to be determined and will be explored in future studies.

## Supporting information

S1 Table Mouse CyTOF antibodies. (DOCX)

S2 Table Human CyTOF antibodies. (DOCX)

S3 Table Human sample demographics. (DOCX)

S1 File Bone marrow cytokine analysis. (PDF)

S1 Data (ZIP)

S2 Data (ZIP)
