# Supplementary material for: Differential immune landscapes in appendicular versus axial skeleton
Source: PLoS One. 2022 Apr 27;17(4):e0267642. doi: 10.1371/journal.pone.0267642 (PMC9045623; doi:10.1371/journal.pone.0267642)
Supplement: S1 Table — (DOCX) [file pone.0267642.s001.docx]

**Supplemental Table 1. Mouse CyTOF antibody panel**

| Antibody | Clone | Isotope |
| --- | --- | --- |
| CD45 | 30-F11 | ^89^Y |
| CD44 | IM7 | ^141^Pr |
| CD11b (Mac-1) | M1/70 | ^142^Nd |
| CD45R (B220) | RA3-6B2 | ^144^Nd |
| CD4 | RM4-5 | ^145^Nd |
| Ly6G | 1A8 | ^148^Nd |
| CD19 | 6D5 | ^149^Sm |
| TCRb | H57-597 | ^150^Nd |
| Ly-6C | HK1.4 | ^151^Eu |
| CD3e | 145-2C11 | ^152^Sm |
| CD161 (NK1.1) | PK136 | ^155^Gd |
| Sca-1 (Ly-6A/E) | E13-161.7 | ^160^Gd |
| TER-119  (Glycophorin A) | TER-119 | ^162^Dy |
| CD8a | 53-6.7 | ^164^Dy |
| CD103 | 2E7 | ^166^Er |
| CD117 (ckit) | 2B8 | ^168^Er |
| FceR1a | MAR-1 | ^169^Tm |
| TCRgd | GL3 | ^172^Yb |
| I-A/I-E (MHC class II) | M5/114.15.2 | ^174^Yb |
| CD197 (CCR7) | 4B12 | ^176^Yb |
| CD11c | N418 | ^209^Bi |
| CD25 (IL-2R) | 3C7 | ^167^Er |
| CD62L (L-selectin) | MEL-14 | ^161^Dy |
| CD69 | H1.2F3 | ^173^Yb |
| cd90 | G7 | ^171^Yb |
| Cytokeratin 8 | 1E8 | ^159^Tb |
| CD29 | HMB1-1 | ^147^Sm |
| CD150 | TC15-12F12.2 | ^165^Ho |
| CD335 ( NKp46) | 29A1.4 | ^175^Lu |
| CD5 | 53-7.3 | ^143^Nd |
| CD43 | S11 | ^153^Eu |
| FR4 | TH6 | ^154^Sm |
| Siglec-F |  | ^170^Er |
