# Supplementary material for: Differential immune landscapes in appendicular versus axial skeleton
Source: PLoS One. 2022 Apr 27;17(4):e0267642. doi: 10.1371/journal.pone.0267642 (PMC9045623; doi:10.1371/journal.pone.0267642)
Supplement: S2 Table — (DOCX) [file pone.0267642.s002.docx]

**Supplemental Table 2. Human CyTOF antibody panel**

| Antibody | Clone | Isotope |
| --- | --- | --- |
| CD45 | (HI30) | ^89^Y |
| CD196/CCR6 | (G034E3) | ^141^Pr |
| CD123 | (6H6) | ^143^Nd |
| CD19 | (HIB19) | ^144^Nd |
| CD4 | (RPA-T4) | ^145^Nd |
| CD8a | (RPA-T8) | ^146^Nd |
| CD11c | (Bu15) | ^147^Sm |
| CD16 | (3G8) | ^148^Nd |
| CD45RO | (UCHL1) | ^149^Sm |
| CD45RA | (HI100) | ^150^Nd |
| CD161 | (HP-3G10) | ^151^Eu |
| CD194/CCR4 | (L291H4) | ^152^Sm |
| CD25 | (BC96) | ^153^Eu |
| CD27 | (O323) | ^154^Sm |
| CD57 | (HCD57) | ^155^Gd |
| CD183/CXCR3 | (G025H7) | ^156^Gd |
| CD185/CXCR5 | (J252D4) | ^158^Gd |
| CD28 | (CD28.2) | ^160^Gd |
| CD38 | (HB-7) | ^161^Dy |
| CD56/NCAM | (NCAM16.2) | ^163^Dy |
| TCRgd | (B1) | ^164^Dy |
| CD294 | (BM16) | ^166^Er |
| CD197/CCR7 | (G043H7) | ^167^Er |
| CD14 | (63D3) | ^168^Er |
| CD3 | (UCHT1) | ^170^Er |
| CD20 | (2H7) | ^171^Yb |
| CD66b | (G10F5) | ^172^Yb |
| HLA-DR | (LN3) | ^173^Yb |
| IgD | (IA6-2) | ^174^Yb |
| CD127 | (A019D5) | ^176^Yb |
| Cell-ID | Intercalator-103Rh | ^103^Rh |
