# Supplementary material for: Differential immune landscapes in appendicular versus axial skeleton
Source: PLoS One. 2022 Apr 27;17(4):e0267642. doi: 10.1371/journal.pone.0267642 (PMC9045623; doi:10.1371/journal.pone.0267642)
Supplement: S3 Table — (DOCX) [file pone.0267642.s003.docx]

**Supplemental Table 3. Human Sample Demographics**

| Sample | Age (yrs) | Gender | Race | Pathology | Medications affecting bone | Cancer history |
| --- | --- | --- | --- | --- | --- | --- |
| 1 | 57.76 | M | White | Degenerative spine | none | none |
| 2 | 60.52 | M | White | Degenerative spine | none | none |
| 3 | 60.87 | M | White | Hip replacement | none | none |
| 4 | 61.92 | M | White | Hip replacement | none | none |
