# Supplementary material for: Differential immune landscapes in appendicular versus axial skeleton
Source: PLoS One. 2022 Apr 27;17(4):e0267642. doi: 10.1371/journal.pone.0267642 (PMC9045623; doi:10.1371/journal.pone.0267642)
Supplement: S1 File — (PDF) [file pone.0267642.s004.pdf]

S1 File. Bone Marrow Cytokine Analysis

	Ossicle A	Ossicle B	Ossicle C	Vossicle A	Vossicle B	Vossicle C
6Ckine/Exodus 2						
Eotaxin	62.95230769	80.66	149.3907692	54.43826087	63.854	20.49
EPO	0	0	0	28.82782609	13.524	30.735
Fractalkine	55.69461538	45.57	50.59461538	38.48	172.06	116.58
G-CSF	24.54538462	17.43	3.870769231	9.346521739	16.73	4.215
GM-CSF	0	0	0	0	2.618	0
IFNβ-1	72.77307692	46.47	72.77307692	53.40869565	24.416	38.355
IFNγ	0.836923077	1.58	1.974615385	1.029565217	5.712	9.345
IL-10	3.713846154	6.82	6.015384615	12.58	40.614	30.66
IL-11						
IL-12p40	0	0.78	0	174.2378261	87.906	145.29
IL-12p70	0	0	0	0	1.82	0
IL-13	0.287692308	0.11	0.026153846	12.40304348	2.478	0.78
IL-15	13.03769231	9.97	16.93461538	21.63695652	39.494	53.46
IL-16	9707.143846	9837.97	7444.195385	4654.455217	4209.226	3029.19
IL-17	0.313846154	0.45	0.392307692	2.91173913	1.652	6
IL-1α	91.72153846	83.75	74.66923077	556.9143478	341.824	364.545
IL-1β	3.086153846	1.07	3.373846154	2.429130435	4.186	13.755
IL-2	12.16153846	14.27	11.99153846	36.08304348	51.156	55.725
IL-20						
IL-3	0.666923077	0.67	0.876153846	0.514782609	0.714	0.675
IL-4	0.431538462	0.26	0.496923077	0.289565217	0.336	0.255
IL-5	0.130769231	0.17	0.51	0	0.63	0
IL-6	3.962307692	3.65	3.596153846	18.27478261	15.904	21.39
IL-7	2.876923077	3.2	3.06	7.496521739	4.676	16.32
IL-9	13.92692308	17.96	25.44769231	170.4091304	55.342	111.15
IP-10	9.493846154	15.21	15.56153846	18.27478261	12.866	16.155
KC	26.59846154	21.63	16.51615385	75.72130435	34.146	33.885
LIF	2.013846154	2.9	2.824615385	1.512173913	2.506	2.415
LIX	98.19461538	110.94	336.2469231	0	0	0
MCP-1	0.470769231	4.34	3.439230769	8.799565217	5.278	5.655
MCP-5	76.57846154	80.62	59.10769231	64.65347826	107.184	93.84
M-CSF	7.754615385	8.65	11.86076923	1.89826087	6.678	6.96
MDC	13.22076923	24.69	31.96	11.90434783	22.218	7.455
MIG	311.0738462	662.36	305.7646154	77.76434783	63.434	27.435
MIP-1B	0	0	0	0	0	0
MIP-1α	0	12.02	0	37.88478261	37.408	28.92
MIP-2	16.22846154	97.19	147.8476923	220.7613043	312.144	459.57
MIP-3α	1.830769231	2.05	1.778461538	5.517826087	3.08	8.805
MIP-3β	44.71	29.13	42.21230769	55.46782609	68.558	49.635
RANTES	6.734615385	15.65	14.71153846	3.667826087	2.94	2.295
TARC	18.12461538	18.89	36.48461538	20.52695652	18.774	20.19
TIMP-1	5592.463846	9644.93	4701.964615	3485.383913	7281.75	4178.295
TNFα	0	0	0	0	0	0
VEGF	23.53846154	15.12	36.23615385	31.88434783	10.248	21.675
